# Single-cell transcriptomics and mouse model phenotyping for biomarker screen of peripheral blood in Huntington’s disease

**DOI:** 10.1038/s41598-026-54412-4

**Published:** 2026-05-26

**Authors:** Paula Martín-Climent, Samanta Ortuño-Miquel, Juan F. Gallego-Serna, Silvia Martí-Martínez, Luis M. Valor

**Affiliations:** 1 Research Laboratory, Dr. Balmis General University Hospital, Alicante Institute for Health and Biomedical Research (ISABIAL), 03010 Alicante, Spain; 2 Present Address: Centre for Nutrition Research, Department of Nutrition, Food Science and Physiology, University of Navarra, 31008 Pamplona, Spain; 3 Centro de Investigación Biomédica en Red de Fisiopatología de La Obesidad y Nutrición (CIBEROBN), Instituto de Salud Carlos III (ISCIII), Madrid, Spain; 4 Unit of Epidemiology and Bioinformatics, Dr. Balmis General University Hospital, Alicante Institute for Health and Biomedical Research (ISABIAL), 03010 Alicante, Spain; 5 Institute of Research, Development, and Innovation in Healthcare Biotechnology in Elche (IDiBE), Miguel Hernández University, 03202 Elche, Spain; 6 Department of Neurology, Dr. Balmis General University Hospital, Alicante Institute for Health and Biomedical Research (ISABIAL), 03010 Alicante, Spain

**Keywords:** Polyglutamine, Blood, PBMC, scRNA-seq, Biomarker, Human, R6/1, Interferon, Biomarkers, Computational biology and bioinformatics, Genetics, Molecular biology, Neuroscience

## Abstract

Transcriptional dysregulation is among the most prominent molecular alterations in Huntington’s disease (HD). It is not confined to the brain but also extends to peripheral tissues and cells, enabling minimally invasive screening strategies to identify transcriptional surrogates of the health status in HD mutation carriers. Nonetheless, transcriptomics approaches have failed to identify consistent candidates from peripheral blood, probably due to the low impact of the HD mutation in the transcriptional profiles of circulating cells, which can be masked by the high cellular complexity of this biofluid. In this study, we applied for the first time single-cell RNA-seq to peripheral blood mononuclear cells (PBMCs) to determine which cells accumulate the most prominent gene expression changes, and therefore, represent potential sources of reliable biomarkers. We observed common transcriptional alterations across different blood cell subtypes, which were partially validated in published bulk transcriptomics datasets. To relate these gene expression patterns with disease progression in the absence of a large cohort of patients, we examined selected candidates in a phenotypically characterized cohort of transgenic R6/1 mice. Among the tested genes, only the variations in the interferon related gene *Irf7* in blood were mildly correlated with motor coordination performance in mutant mice. Notably, striatal *Irf7* expression did not show such phenotypical correlation in the same individuals. Overall, transcriptional-based changes in peripheral blood can be linked to HD but they are mild and not apparently confined to particular cellular subpopulations. In conclusion, dissection of individual blood cells in combination with subsequent validation in mouse models emphasizes the challenges in obtaining clinically relevant biomarkers in HD.

**Supplementary Information:**

The online version contains supplementary material available at 10.1038/s41598-026-54412-4.

## Introduction

Huntington’s disease (HD) is a highly disabling condition with lethal consequences that is caused by an aberrant expansion of CAG triplets in the Huntingtin (*HTT*) gene. While waiting for an effective therapy, HD mutation carriers require specific biomarkers to report their actual health status, to predict the onset and progression of the diverse manifestations of symptoms, and to evaluate the response to therapeutic regimes. However, our current knowledge of biomarkers has not been effectively incorporated into routine HD clinical management^1^. Regarding potential biomarkers sources, peripheral blood obtained through venipuncture is among the most preferred option due to its minimal invasiveness, high feasibility, and broad applicability. Moreover, it has the potential to inform about both systemic and brain-derived pathological perturbations, since neural by-products can be released into the bloodstream.

We and others have extensively reviewed the relevance of transcriptional dysregulation in HD^2–4^, which is an early phenomenon that is not restricted to the brain regions most affected by the *HTT* mutation (e.g., basal ganglia and associated cortical areas) but can also be detected peripherally (e.g., liver, muscle, adipocyte tissue, blood, skin), further supporting the non-neuronal clinical symptomatology of HD (reviewed in^1^). Since the beginning of modern HD research, transcriptomics technologies have been applied to identify gene expression changes in the peripheral blood of mutation carriers. The goal has been to link these accessible and quantifiable variations to different disease stages that, ideally, can precede the onset of declared symptomatology in sufficient time to support decision-making^1^. However, the retrieval of clinically relevant candidates has proven to be difficult. Despite using validation cohorts within individual studies, the proposed candidates were, at best, partially confirmed in a few independent reports, without being consistent across studies. This is exemplified by efforts to integrate newly generated datasets with previous findings, in which overlapping genes were mainly detected in a pair-wise fashion^5,6^. These results indicated that transcriptional changes in HD blood are not as pronounced as in the brain and cannot overcome the interindividual variability and demographic variation inherent to human populations. This situation is exacerbated by the usage of diverse transcriptomics platforms and analytical procedures that hinder direct comparisons across studies.

Exploration of transcriptional dysregulation in blood is still fully justified because the expression of mutant HTT (mHTT) has been detected in both innate and adaptive immune cells^7^ and circulating proinflammatory factors (e.g., IL-6, IL-8, TNF-α) are increased according to disease stage^8–10^. In this context, the high cellular heterogeneity of blood may be masking the nuclear effects of *HTT* mutation, which can be critical if such changes are only prominent in certain cell subpopulations. Single-cell RNA-seq (or its single-nucleus variant snRNA-seq) is widening our understanding of HD pathogenesis through dissecting defects across multiple cell types to illustrate the unforeseen cellular heterogeneity even in seemingly homogeneous subpopulations^11–14^. Although this costly technology is still not suitable for large cohort studies, we applied for the first time single-cell transcriptomics (scRNA-seq) to few peripheral blood samples from symptomatic volunteers, in order to determine which disease-specific cell variants are more susceptible to contain reliable biomarkers (i.e., which cell subpopulation(s) accumulate most of the disease-dependent molecular alterations) and how biomarker candidates behave across different cell types. Thus, isolation of specific subpopulations can augment detection sensitivity (as reported for myeloid cells from patients and mouse models^15,16^). In other words, this knowledge may lead to further exploration of specific cellular targets using more affordable techniques in the clinical settings.

We complemented this approach by investigating the correlation between gene expression disruption and the pathological phenotype in a mouse model, which, in our case, was more feasible than analysing clinical data from large cohorts. Recruiting sufficient subjects to achieve reliable statistical power is challenging for rare disorders, not normally supported by sample collections from initiatives such as the Enroll-HD (https://www.enroll-hd.org) for biomarker research. In addition, the use of animal models enables the direct comparison between brain and peripheral alterations, assuming that most alterations are conserved across species (as it happens with transcriptional dysregulation in brain^17,18^).

This work is not intended to provide biomarkers of prognosis but, instead, it is a proof-of-concept study: i) to assess the utility of single cell transcriptomics for the proposal of novel candidates in biomarker screens, and ii) to evaluate the use of phenotypically characterized animal models for the validation of novel biomarker candidates.

## Materials and methods

### Human samples

The study was conducted according to the guidelines of the Declaration of Helsinki, following the national and regional laws and regulations concerning biomedical research on human samples, personal data protection and the use of biobank services, after approval by the local ethics committee: Comité de Ética de Investigación Clínica con medicamentos (CEIm) del Hospital General Universitario Dr. Balmis (HGUDrB), reference number PI2021/058, 12^th^ November 2021. Samples and clinical data were provided by the Biobank of ISABIAL which is adhered to the Spanish National Biobanks Network and integrated in the Valencian Biobanking Network, following standard operating procedures. All participants gave written informed consent.

Human volunteers were recruited through the Service of Neurology of the HGUDrB, and consisted of 4 controls (without the HD mutation) and 4 HD symptomatic patients, with equal representation of both sexes in each condition (2 women and 2 men) and balanced age at sampling (median (min–max) = 52.0 (51–76) in controls, median (IQR) = 57.5 (42–62) in patients). The number of CAG repeats in the expanded allele was 43.5 (41–46) in the HD group. The stage of HD according to the Total Functional Capacity (TFC)^19^ was 1 for all controls and 4–5 for all symptomatic patients.

Human blood samples were collected in EDTA-K2 Vacutainer tubes (BD) for isolation of peripheral blood mononuclear cells (PBMC) after a density gradient centrifugation using Pancoll human, 1.077 g/mL (PAN-Biotech) following this procedure: 1,300xg 15 min, followed by a wash with 0.1 M PBS 1,000xg 5 min. Cells were immediately cryopreserved in two aliquots per donor using the Demonstrated Protocol CG00039 RevD “Fresh Frozen Human PBMC for scRNA-seq” (10xGenomics), which was previously validated in our laboratory to preserve the integrity of total RNA.

### Single-cell RNA-seq assays

**Fig. 1:**
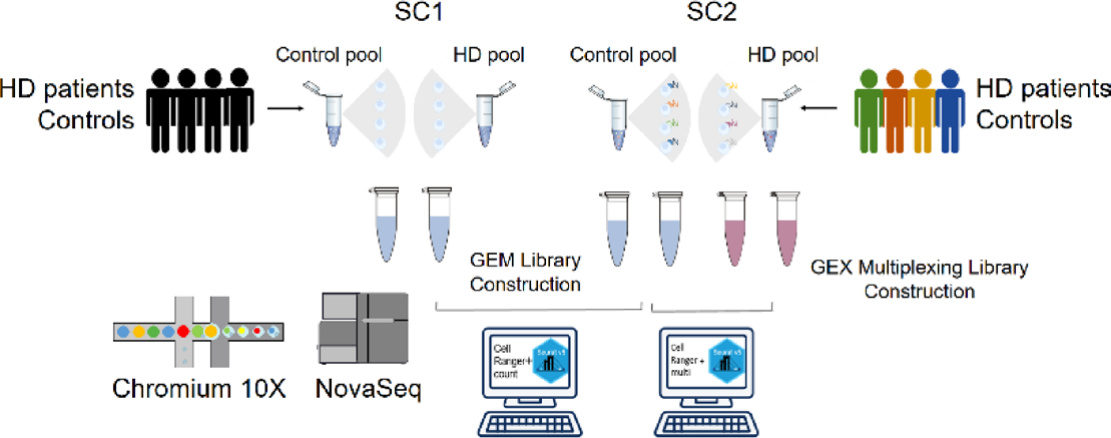
Workflow of the single-cell RNA-seq assays.

The workflow of these assays is outlined in Fig. 1. The samples were used for a first scRNA-seq experiment (SC1) using the Chromium Next GEM Single Cell 3ʹ Reagent Kits v3.1 (Dual Index) protocol with Feature Barcode technology for Cell Multiplexing (10 × Genomics). We created two pools (CTRL and HD) from samples; briefly, one of the aliquots of each cryopreserved sample was thawed and washed following the Demonstrated Protocol CG00039 RevD “Fresh Frozen Human PBMC for scRNA-seq” (10xGenomics), and subsequently sorted in a FACSAria III (Becton Dickinson) to remove cell debris and platelets based on size and scatter properties (Supplementary Fig. 1) and to collect equal number of cellular events (~ 4,500 / sample) that was used to create the CTRL and HD pools. Both pools were processed with the Chromium Next GEM Single Cell 3ʹ Reagent Kits v3.1 (Dual Index) protocol to obtain single cell barcodes for sequencing. Then, we built the DNA libraries and assessed their quality according to the aforementioned protocol. Another experiment was conducted (SC2) with the same samples using the same protocol with the exception of including a prior labelling procedure for each individual sample using Cell Multiplexing Oligo (CMO) tags linked to cell-associated barcodes. The resulting libraries were sequenced in Illumina NovaSeq 6000 system at the Genomics Unit of the Cabimer to achieve 25,000 reads/cell (3’-GEX libraries) and 5,000 reads/cell (CMO libraries).

### Bioinformatics analysis and statistical analysis

For alignment with the human reference genome GRCh38, we used the 10 × Genomics Cell Ranger (v7.2) software. Whereas the ‘cellranger count’ pipeline was used for both experiments, the ‘cellranger multi’ pipeline was also used in SC2 to track single transcriptomes from each individual, adjusting the ‘min-assignment-confidence’ parameter to 0.7 to assign more cells to the tags.

Subsequent analyses were conducted using the “Seurat” package in R (v4.0.4)^20^. The single-cell transcriptomes were filtered according to the following quality control metrics: number of unique genes (UMIs) detected per cell (between 800 and 6,000), total number of molecules (reads) within a cell (between 2,000 and 30,000), and percentage of reads mapped to the mitochondrial genome (< 15%). The data were then normalized with the “NormalizeData” function applying log-normalization by default. The “scDblFinder” package was used to identify and remove potential doublets (cells with an estimated doublet density score per cell > 3) from the dataset. Following doublet filtering, highly variable features were identified (“FindVariableFeatures”), scaled (“ScaleData”) and subjected to Principal component analysis (PCA) (“RunPCA”) in which a dimensionality reduction and clustering pipeline was then applied using the first 20 Principal Components. Cell–cell neighbourhoods were computed using “FindNeighbors” function, and clustering was performed using the Louvain algorithm via “FindClusters” with a resolution parameter set to 0.5.

To explore cellular heterogeneity, we used markers described in “Azimuth”^21^ to identify which cluster corresponded to each cell type. After trial and error, we opted for grouping minor cell subpopulations that shared cell-specific markers, leading to these groups: CD14^+^ monocytes (*S100A9, CTSS, S100A8, LYZ, VCAN, S100A12, IL1B, CD14, G0S2, FCN1*), other myeloid cells that included dendritic cells (*CTSS, FCN1, NEAT1, LYZ, PSAP, S100A9, AIF1, MNDA, SERPINA1, TYROBP, CD74, HLA-DPA1, HLA-DPB1, HLA-DQA1, CCDC88A, HLA-DRA, HLA-DMA, CST3, HLA-DQB1, HLA-DRB1*), CD4^+^ T-cells (*IL7R, MAL, LTB, CD4, LDHB, TPT1, TRAC, TMSB10, CD3D, CD3G*), other T-cells (*CD3D, TRDC, GZMK, KLRB1, NKG7, TRGC2, CST7, LYAR, KLRG1, GZMA*), NK-cells (*NKG7, KLRD1, TYROBP, GNLY, FCER1G, PRF1, CD247, KLRF1, CST7, GZMB*), naïve B-cells (*IL4R, TCL1A, YBX3*) and active B-cells that included intermediate and memory cells (*COCH, SSPN, LINC01781, NEK6, LINC01857*). Such grouping also enhanced the statistical power of the differential expression analysis within each cell subpopulation (see below). As an additional quality control of our datasets, we also examined the distribution of markers related to plasmablasts (*IGHA2, MZB1, TNFRSF17, DERL3, TXNDC5, TNFRSF13B, POU2AF1, CPNE5, NT5DC2*) as too many short-lived plasmablasts might indicate unrelated infections or vaccinations challenges that might distort analysis interpretration, and platelets (*PPBP, PF4, NRGN, GNG11, CAVIN2, TUBB1, CLU, HIST1H2AC, RGS18, GP9*) to assess the efficiency of our cell sorting procedure. Gene expression patterns of independent cell-specific markers were visualized with the “DotPlot” function from “Seurat” (v5.3.0), splitting by the genotype variable.

Trajectory inference and pseudotime analyses were performed in R (v4.5.0). Cell trajectories were reconstructed with “slingshot” (v2.16.0) on defined clusters^22^. UMAP embeddings were obtained from the “reducedDims” slot, plotted using the “viridis” colour scale (v0.6.5) to represent pseudotime, and the inferred lineages were overlaid. Gene dynamics along pseudotime were modelled using “tradeSeq” (v1.22.0) with generalized additive models (GAMs)^23^. The number of knots was determined automatically and consistently set to 10 across all clusters. Markers of immune cell differentiation stages were found among the genes defining the trajectories (retrieved with “associationTest” function with Benjamini–Hochberg correction).

Within each cellular subpopulation, the differential expression analysis was performed between CTRL and HD using the “FindMarkers” function of the “MAST” package (https://github.com/RGLab/MAST/). Due to different representation of donors despite pooling the same number of sorted cells from each individual for the oil encapsulation process, we included sex as a latent variable to minimize the contribution of sex chromosome-linked genes (see Results and discussion for the uneven distribution of the female marker *XIST*). We considered as differentially expressed genes (DEGs) between controls and patients in each defined subpopulation when the adjusted p-value ≤ 0.05 (using the Benjamini–Hochberg method by default). With these results, we considered those DEGs as “Specific” for a particular cellular subpopulation if they were not found in any other pair-wise comparison between control *vs*. patient in the rest of subpopulations (detected in Venn diagrams (https://bioinfogp.cnb.csic.es/tools/venny/, https://bioinformatics.psb.ugent.be/webtools/Venn/); on the contrary, they were considered as “Shared” if found in at least two pair-wise comparisons.

DEGs were compared with those already reported for bulk peripheral blood and brain as they appeared in the Supplementary Tables of the corresponding publications (see Results and discussion for further details). To determine whether our DEGs were expressed in human brain, we used the external scRNA-seq datasets from GSE152058 consisted of human caudate nuclei^11^. First, these datasets were processed using the same pipeline applied to our in-house data, using the cellular clusters proposed in the original publication. The “FindAllMarkers” function identified those genes more overexpressed in each cluster (log_2_ fold change > 1, adjusted p-value < 10^–99^). Then, the resulting gene lists were compared with the whole list of DEGs from PBMCs.

Additional statistical analyses (Mann–Whitney U-tests, Spearman’s rank-order correlation coefficients) were conducted in native R environment (v4.4.0).

### Behavioural and gene expression analysis of R6/1 strain

We previously showed in the transgenic B6.Cg-Tg(HDexon1)61Gpb/J strain (*aka* R6/1^24^) (originally provided by the Jackson Laboratory and bred at the facilities of Servicio de Experimentación y Producción Animal of the Cádiz University) that transcriptional dysregulation of HD-related genes showed tissue-specific associations with certain pathological phenotypical traits (see^25^ for further details). For the current study, we reanalyzed the rotarod and weight measurements made in 11 and 13 weeks-old animals and reused the striatal and blood samples immediately extracted five days after the behavioural battery (14 weeks-old)^25^. All procedures (including housing conditions, experimental protocols, phenotyping, animal handling and euthanization by cervical dislocation in individuals < 26 g of body weight) are fully described in our previous publication and were approved by the corresponding ethical committee (Comité de Ética de Experimentación Animal—Órgano Habilitado de la Universidad de Cádiz and authorized by the Dirección General de la Producción Agrícola y Ganadera de la Junta de Andalucía) according to European, national and regional regulations. This study was in compliance with the ARRIVE guidelines.

Quantitative PCR was performed in QuantStudio 12 K Flex (Thermo Fisher, Madrid, Spain) using HOT FIREpol EvaGreen qPCR Mix Plus 5X (ROX) (Solis Byodine, Tartu, Estonia). The PCR cycling conditions were as follows: 95 °C for 15 min and 40 cycles of 95 °C for 15 s, 60 °C for 20 s, 72 °C for 20 s. Each independent reaction was normalized to the level of *Eef2*^26^, and the relative quantitative values were calculated according to the 2^−ΔΔCT^ method. The sequences of all primer pairs are: *Dse*, 5’-GCCTTACTCACTGGAAGTCT-3’ and 5’-TGCTTAGAACTTGCTTGGTC-3’; *Ifit2*, 5’-TTTGACACAGCAGACAGTTA-3’ and 5’-TCCAGTGACTCCTTACTCGT-3’; *Ifitm1*, 5’-CTGAGATCTCCACGCCTGAC-3’ and 5’-CCAGTCGTATCACCCACCAT-3’; *Irf7*, 5’-AAGCATTTCGGTCGTAGGGAT-3’ and 5’-AGTTCGTACACCTTATGCGGA-3’; *Mx1*, 5’-GGTCCAAACTGCCTTCGTAAA-3’ and 5’-CGGATCAGGTTTTCAGCTTCC-3’; *Rsad2*, 5’-AGGACAGGGGTGAATACTTGG-3’ and 5’-CACGGCCAATCAGAGCATTAA-3’; *Samsn1*, 5’-AGGAGAAACACCAAAAACCG-3’ and 5’-ATTCCGAAAACGGTCAAAAT-3’.

## Results and discussion

### scRNA-seq technology detected mild changes in peripheral blood of symptomatic patients that were partially confirmed in published bulk transcriptomics studies

**Fig. 2:**
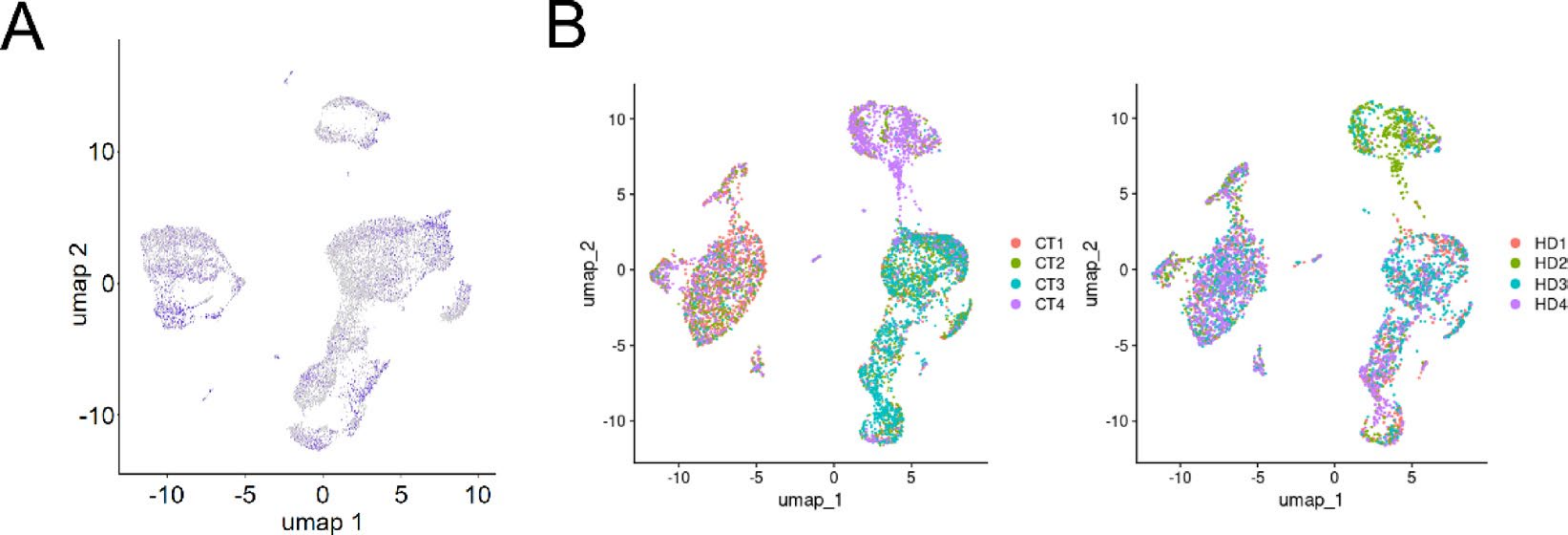
Different contribution of donors to cellular clusters. (**A**) UMAP with the distribution of *XIST* expression (SC1). (**B**) UMAP with the distribution of cells per donor, as tagged using CMOs prior to sample pooling (SC2).

To determine the most altered PBMC subpopulations in HD patients, we performed a first scRNA-seq assay (SC1) comparing two pools of cells from symptomatic donors (HD) and controls without the CAG expansion (CTRL), balanced in sex and age (Fig. 1). After assessing quality (Supplementary Fig. 1), we retained 7761 cells from the CTRL pool and 6788 cells from the HD pool, which contained a mean ± SD of transcripts per cell of 9019.7 ± 5103.9 and 8516.4 ± 4922.6, respectively. Despite pooling the same number of cells from each donor (two women and two men), the results of a preliminary differential expression analysis were jeopardized by genes belonging to chromosomes X (e.g., *XIST*, *RPS4X*, *RPL36A*, *TMSB4X*) and Y (e.g., *RPS4Y1*, *USP9Y*, *DDX3Y*, *UTY*, *EIF1AY*, *PRKY*) (see the example of *XIST* expression across clusters in Fig. 2A). This observation indicated that upstream processing and analysis (e.g., storage and sample preparation, cellular encapsulation and labelling, and quality filtering of the transcriptomes) might produce stochastic effects, possibly amplified by the technical noise characteristic of low starting material in single-cell transcriptomics^27^. Consequently, this may lead to differential contributions (and distributions) of cells from each donor across the resulting clusters. To confirm this, we performed a second scRNA-seq assay (SC2) using the same samples and the same batch of reagents, with the only difference being the tagging of individual samples using CMOs prior to pooling for single-cell labelling (Fig. 1). As observed in Fig. 2B, the distribution of CMOs was also uneven across clusters in both CTRL and HD pools, which was reminiscent of the *XIST* gene distribution (Supplementary Fig. 2). For this reason, we considered sex as an additional variable to minimize the influence of sexual chromosomes in subsequent analyses.

**Fig. 3:**
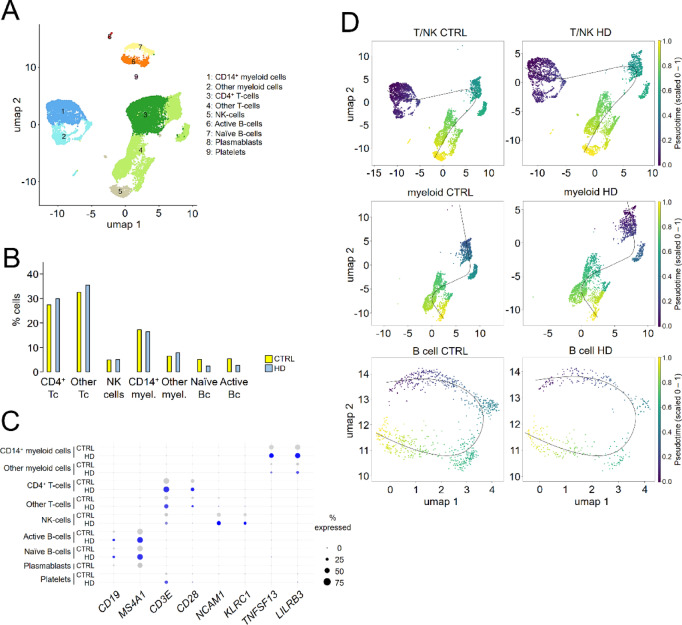
General results of single-cell RNA-seq assay in PBMCs from HD patients and control donors. (**A**) UMAPs showing the PBMC subpopulations from SC1. (**B**) Percentage of cells for each subpopulation from the total that passed the quality filters for each pool of controls (CTRL) and patients (HD). (**C**) Dot plot of cell-specific markers across the defined cellular clusters in both pools. (**D**) UMAPs of CTRL and HD cells across the global trajectories defined in major cell clusters, scaled from 0 to 1.

Taking advantage of the high quality of SC1, in which filtered cells from CTRL and HD pools showed highly similar distributions of UMIs and total reads per cell (Supplementary Fig. 1), we clustered the individual transcriptomes of this assay into relevant cell subpopulations (T-cells, NK-cells, myeloid cells, and B-cells). Cellular subtypes with low abundance of cells were grouped according to the distribution of common markers to reduce redundancy while preserving cellular identity (Fig. 3A). In this way, we reduced data complexity while increasing the statistical power of downstream differential expression analysis. We did not observe substantial differences in cell proportion across clusters between both genotypes (Fig. 3B). The resulting proportions (75.5% of lymphocytes consisted of 82.7% of T cells, 10.7% of B cells, 6.6% of NK cells, and 24% of myeloid cells including monocytes and dendritic cells) fell within the expected range^28^; whilst only a very small fraction of unwanted cell types (plasmablasts and platelets, 0.4% and 0.1%, respectively) was obtained. We confirmed the resulting cell subpopulation annotation by plotting specific markers for T-cells (*CD3E*, *CD28*), B-cells (*CD19*, *MS4A1*), NK cells (*NCAM1*, *KLRC1*) and macrophages/monocytes (*TNFSF13*, *LILRB3*), not used for cluster identification (Fig. 3C). Based on the UMAP, we performed a trajectory analysis in the three major clusters of cells (all T and NK cells, all myeloid cells and all B-cells) with sufficient number of cells to reflect the maturation and differentiation dynamics of peripheral immune subpopulations in the SC1 experiment: as observed in Fig. 3D, cell distribution across global trajectories was highly similar between genotypes.

**Fig. 4:**
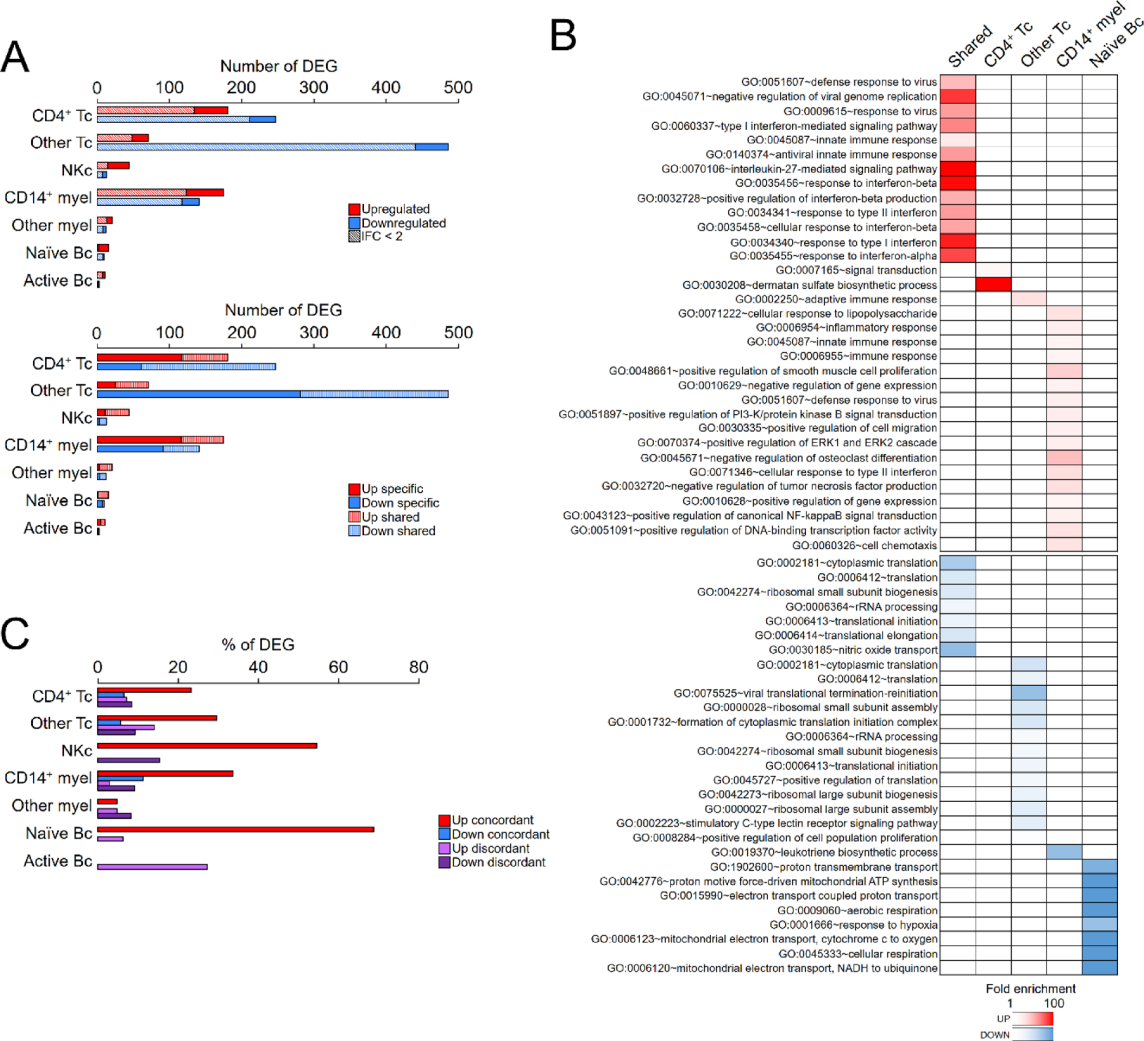
Differential expression analysis in PBMC subpopulations between HD patients and control donors. (**A**) Number of differentially expressed genes (DEGs) between CTRL and HD in each subpopulation and assay (adjusted p-value < 0.05, MAST). Upregulated and downregulated genes in patients’ cells compared to controls’ cells are shown; “Specific” refers to significant genes that are only found in this subpopulation whereas “Shared” refers to genes observed in at least two subpopulations. (**B**) Significant Gene Ontology (GO) enriched terms (FDR < 0.05) obtained from specific and shared DEGs for each direction of change (UP and DOWN). No significant term was retrieved in “Other myeloid” and “Active Bc”. (**C**) Percentage of DEG in each cell subpopulation that was reported in at least one publication^5,6,15,30–33^. “Concordant” and “discordant”, in the same or opposite direction of change, respectively.

Next, we conducted the differential expression analysis between control *vs*. patient cells in each cell subpopulation depicted in Fig. 3A. Non-CD4^+^ cells were apparently the most affected cellular subpopulation (Fig. 4A), although the magnitude of changes were minor (fold change < 2) and further confirmation in independent samples is required. Although important fractions of DEGs in patients related to controls were apparently specific of the defined cell subpopulations (i.e., only appearing in a single pair-wise comparison between controls and patients), ~ 52% of upregulated genes and ~ 35% of downregulated genes were detected in more than one population (Fig. 4A). Among the most frequent DEGs across cell subpopulations (appearing at least in five pair-wise comparisons between controls and patients), we found the following genes: the eukaryotic translation initiation factor kinase *EIF2AK2*, the endoplasmic reticulum aminopeptidase *ERAP2*, the epithelial stromal interactor *EPSTI1*, the interferon-induced *IFI44*, *IFI44L* and *IFIT3*, the ubiquitin-like modifier *ISG15*, the mitochondrial-like *MTRNR2L12,* the dynamin GTPases *MX1* and *MX2*, and the interferon-induced apoptotic *XAF1* as upregulated genes, together with the haemoglobin *HBB*, the ribosomal *RPS26* and the adhesion G-protein coupled receptor *ADGRG7* as downregulated genes in patients. No information has been found regarding these genes in HD, although genetic variants of *EIFAK2* have been linked to dystonia^29^.

Unsurprisingly, upregulated genes were mostly related with immune response (innate, interferon-mediated, viral), showing minor functional specificity (e.g., chemotaxis in CD14^+^ monocytes) (Fig. 4B). Downregulated genes were mainly enriched in protein translation and ribosomal biogenesis, which is intriguing given that these processes are highly energy-demanding and require fine-tuned regulation under T cell activation and subsequent proliferation^34–36^, and moreover, their components are upregulated in B-cell maturation, probably to face antibody production^37^. Intriguingly, recent reports indicate that ribosome stalling is common in the HD striatum^38–40^ and leads to the accumulation of ribosomal-related proteins in polysomes^41,42^. Whether the observed transcript downregulation obeyed to a homeostatic response to restore ribosome functionality remains to be explored. Other enriched functions were mitochondrial genes in naïve B-cells and leukotriene metabolism in CD14^+^ monocytes; the latter pathway produces important mediators of inflammatory and allergic responses that can be therapeutically targeted to modulate neuroinflammation and oxidative stress in neurodegenerative conditions, including striatal neurotoxicity^40,43–45^. Again, whether the downregulation of components associated with these processes in HD PBMCs was part of a homeostatic response to limit the hyper-reactivity reported in peripheral immune cells from HD patients and mouse models^7,8,15,16,46^ deserves further attention.

Our results indicated that several changes were retrieved across multiple cell types (Fig. 4A); these overlaps might be larger because different subpopulation sizes might have different statistical power. Such degree of unspecificity should be detectable in bulk transcriptomics studies. Indeed, the percentage of DEGs in scRNA-seq also found in any of these studies varied by an average of ~ 13% (Fig. 4C). More precisely, overlapping upregulated genes were more frequent and concordant than overlapping downregulated genes (Fig. 4C and Table 1), the most frequent genes being interleukin *IL1B* and phospholipid scramblase *PLSCR1,* appearing in three of the reported studies according to the statistical thresholds from the original publications. Regarding the changing genes, ~ 22% of the upregulated genes were not cell-specific according to our cut-offs, which justified their detection in whole blood experiments. Considering differences in statistical thresholds, cell-type specificity and technology platforms of these studies, these comparisons should be regarded as supportive evidence rather than definitive validation.

**Table 1:** Common differentially expressed genes (DEGs) between scRNA-seq results of this study and those published from bulk transcriptomics. ^1^unadjusted p-value < 0.0005; ^2^adjusted *p*-value < 0.05; ^3^unadjusted *p*-value < 0.05; Bold, common DEGs in at least two published datasets in any cell subtype. SAGE, Serial Analysis of Gene Expression.

Cells	Up	Down	Source	Method	References
CD4^+^ Tc	*ANXA1*		Whole blood	µarray^1^	^30^
CD14^+^ myel	*CXCL8, EGR1, ****IER3***, ***IL1B***		Whole blood	µarray^2^	^31^
CD4^+^ Tc	*CD82*, ***FUT7****, S100A11*	*SFMBT2*	Whole blood	SAGE^2^	^6^
Other Tc		*GNPTAB*			
CD14 + myel		*SUN2*			
Shared	*NIBAN1*	***AHNAK****, RASA3*			
CD4^+^ Tc	*CLIC1, ****DCP2****, ****DSE****, ****GBP1****, ITGB1, TBK1, ZC2HC1A*	*CENPP, DCXR, DNMT3A, KCNAB2, PCIF1*	Monoc. culture	RNA-seq^3^	^15^
Other Tc	*PTPRM, RESF1*	*ANTKMT, COX4I1, DDOST, EEF2, LIMD2, PHGDH, RBM38, REPIN1, RPL10, RPS2,SLC25A3, TPI1*			
NKc	*CENPK, CX3CR1, DTX3L*				
CD14^+^ myel	***ANPEP****, CCL3, CCL3L1, CDC42EP2, DNAJA1, DUSP6, GMPR, ****IER3****, ****IL1B****, OTOF, PNPT1, RNASE2, SPATS2L, ****TNFAIP6****, WSB1*	*ARHGAP45, CARS2, FAM20C, ITGB2, LAMP1, LIPA, LSP1, SH3BGRL3*			
Shared	***EIF2AK2****, ****IFI44L****, IFIH1, ****IFIT2****, ****OASL****, PID1, ****PLSCR1****, SAMD9, SAMD9L, ****SAMSN1****, SP100, XAF1*	*ENO1, GPX4, ITGA6, MTUS1, NOP53, RPL3, SLC27A4, SUMF1, TSPO*			
CD4^+^ Tc	***DCP2****, ****DSE****, ****FUT7****, RORA*	*DDX11*	Whole blood	RNA-seq^3^	^32^
Other Tc		*PPM1L, SRSF5*			
CD14^+^ myel	*AVPI1, BTG1, CD86, CLEC6A, EREG, KIAA1958, LRRC8B, ****OAS3****, ****SIGLEC1***				
Shared	*C1orf56, ****EIF2AK2****, ****HERC5****, IFI27, ****IFI6****, IFITM1, ****IRF7****, KYNU, ****MX1****, OAS1, ****PLSCR1****, ****RSAD2****, ****SAMSN1****, TNFSF13B*	***AHNAK***			
CD4^+^ Tc	*EIF1AY, ****GBP1****, PRKY, PSMB9*		Whole blood	RNA-seq^2^	^5^
Other Tc		*EIF1AX, NELL2*			
NKc	*IRF9*				
CD14^+^ myel	***ANPEP****, APOBEC3A, CCR1, DOCK4, FOLR3, ****IL1B****, ****OAS3****, ****SIGLEC1****, ****TNFAIP6***	*MTSS1*			
Shared	*CMPK2, EPSTI1, ****HERC5****, IFI44, ****IFI44L****, ****IFI6****, IFIT1, ****IFIT2****, IFIT3, ****IRF7****, ISG15, LY6E, ****MX1****, MX2, ****PLSCR1****, RPS4Y1,****RSAD2****, STAT1*				

### Potential validation of novel biomarkers using a controlled and phenotypically characterized mouse model

**Fig. 5:**
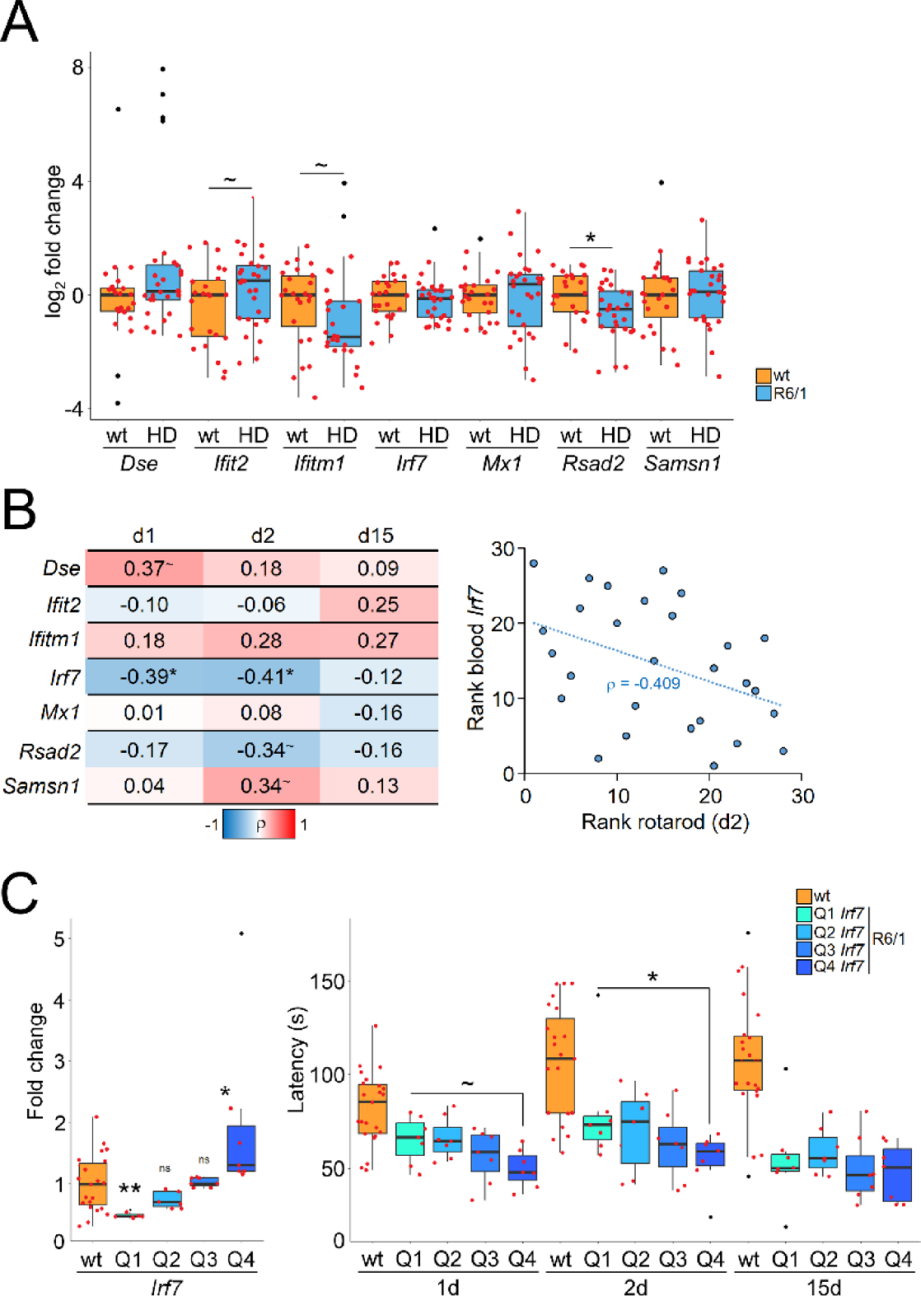
Correlation between blood gene expression and motor performance in the R6/1 mouse model. (**A**) Box-and-whisker plots showing the fold changes in mutant mice (HD, n = 28) compared to wild-type littermates (wt, n = 23) for the indicated genes in blood. *, *p*-value < 0.05; ~ , *p*-value < 0.1 (Mann–Whitney U-test). (**B**) Left, heatmap of Spearman correlation coefficients (ρ) between the examined transcripts in blood from R6/1 mice and rotarod performance at day 1 (d1), 2 (d2) and 15 (d15) (see^25^) (*, unadjusted *p*-value < 0.05; ~ , unadjusted *p*-value < 0.1). Right, correlation between peripheral *Irf7* expression and latency on the rotarod. The dotted line represents the linear regression line. (**C**) Box-and-whisker plots showing the rotarod performance (measured as latency in s) for the groups of animals (right) according to the expression changes of *Irf7* in blood (left); i.e., mice were grouped into four quartiles (Q1–Q4) based on *Irf7* expression levels in blood, where Q1 represents the lowest and Q4 the highest expression. ns, not significant; *, *p*-value < 0.05; **, *p*-value < 0005, Mann–Whitney U-test referred to wt (left) and between quartiles Q1 and Q4 (right).

Next, we examined whether the described peripheral blood alterations in HD patients could be linked to disease progression as potential biomarkers. To this aim, we took advantage of a previous phenotypical characterization of early symptomatic R6/1 mice and accompanying samples from the same animals^25^. Briefly, mice were subjected to a battery of behavioural tests which included measurements of certain phenotypical traits when animals were 11 and 13 weeks old to assess progression. Samples were collected five days after the last measurements (avoiding the potential influence of genes related with handling stress) to examine brain changes in gene expression that could be correlated with the manifested pathological traits across individuals; for example, the altered expression patterns of striatal *Penk*, *Plk5* and *Itpka* genes were mildly correlated with poor performance in the accelerated rotarod and with weight loss^25^. Together with the brain tissue dissections, we also collected blood samples of the same reported animals (28 R6/1 mice and 23 wild-type littermates) to validate whether the gene expression patterns observed in total blood and PBMCs of patients might be correlated with the phenotypical traits measured in mouse. For these assays, we selected genes that were found to be expressed in the most abundant PBMC cell types (Table 1). Of these, only *Ifit2* exhibited a trend towards upregulation, whereas other genes were downregulated in R6/1 samples (most prominently *Rsad2*), indicating that there were prominent species-specific differences at the peripheral level (Fig. 5A). An alternative explanation is that transcriptional changes in human blood were too mild, as evidenced by the extensive use of unadjusted *p*-values in global-wide studies^32,47,48^, to be reliably reproduced in mouse models.

In any case, gene expression may still exhibit relevant differences across individuals that might be reflected at the phenotypical level. Focusing on the rotarod performance, the most well-known paradigm to assess impaired motor coordination in HD mouse models, motor impairment was significantly worsened as *Irf7* became upregulated in mutant mice, observed in the linear correlation analysis (Fig. 5B) and after comparing R6/1 animals classified according to quartiles of *Irf7* expression (Fig. 5C). IRF7 is a member of the interferon regulatory transcription factors family that recognizes the interferon (IFN)-stimulated response element (ISRE) in the DNA to mediate the response of IFN type I and III in viral infections but also in autoimmune diseases and cancer^49,50^ . However, its precise role in HD has not been investigated yet. This correlation was significant when comparing the rotarod latencies at an early stage (11 weeks old) which was later lost (13 weeks old) due to a rapid decline in this phenotypical trait (see^25^ for further details). This resulted in the leverage of this behaviour in all mutant mice independently of the *Irf7* alteration (Fig. 5B-C) or other striatal genes^25^. No correlation was observed with weight loss, another hallmark of HD (Supplementary Fig. 3). Overall, these results suggested that peripheral transcriptional changes were mildly linked to measurable manifestations of the disease in mice (but note that significance is lost when *p*-values were adjusted for multiple testing) and, therefore, more systematic screenings will be required to find stronger associations.

### Blood alterations in gene expression can be found in the brain

**Fig. 6:**
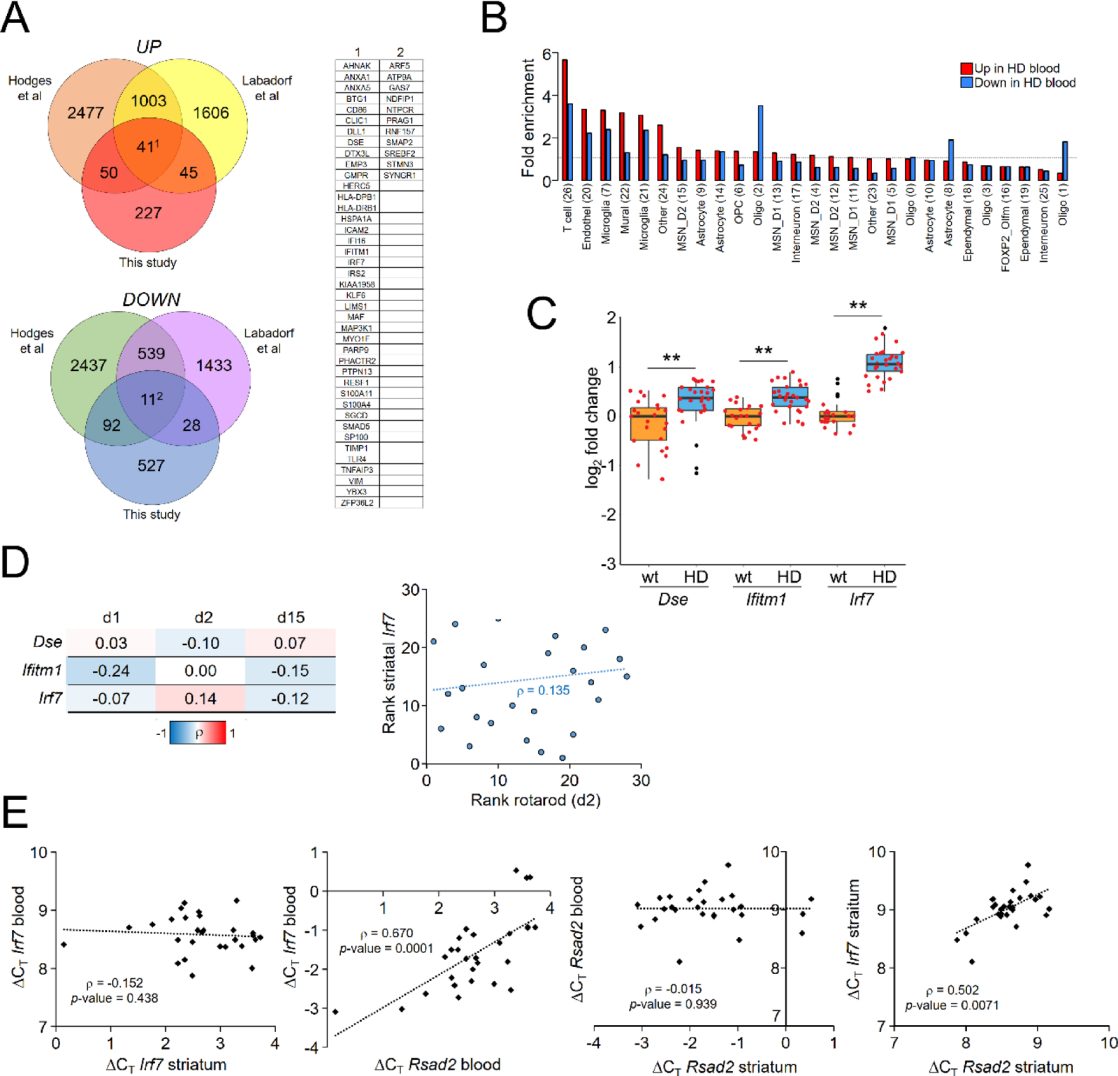
Analysis of blood transcriptional counterparts in the brain of patients and R6/1 mice. (**A**) Venn diagram with the overlapping DEGs between scRNA-seq data from patients’ PBMCs (“This study”) and published bulk RNA-seq data from post-mortem patients’ brains (“Hodges et al.”^18^ and “Labadorf et al.”^51^). Besides, table showing the overlapping genes in all studies following the numbering of the Venn diagram. (**B**) Fold enrichment of peripheral DEGs expression across cell subtypes as defined^11^ for human caudate nucleus (cluster number in parentheses). (**C**) Box-and-whisker plots showing the fold changes in mutant mice (HD, n = 28) compared to wild-type littermates (wt, n = 23) for the indicated genes in striatum. **, *p*-value < 0.005, Mann–Whitney U-test. (**D**) Left, heatmap of Spearman correlation coefficients (ρ) between the examined transcripts in striatum from R6/1 mice and rotarod performance at day 1 (d1), 2 (d2) and 15 (d15)^25^. Right, correlation between striatal *Irf7* expression and latency on the rotarod. The dotted line represents the linear regression line. (**E**) Gene expression is represented as ΔC_T_ (C_T_ gene – C_T_ *Eef2*) and correlation as Spearman correlation coefficient (ρ) and associated unadjusted *p*-value.

To establish a potential link between brain events and altered patterns of peripheral biomarkers, we investigated whether the DEGs defined in the scRNA-seq approach were found in published datasets obtained from post-mortem caudate nuclei^18^ and prefrontal cortex^51^ (unadjusted *p*-value < 0.001 and adjusted *p*-value < 0.05, respectively, according to the original publications). Doing so, it was revealed that ~ 38% of upregulated genes and ~ 20% of downregulated genes in patients’ PBMCs were also disrupted in the same direction of change in patients’ postmortem brain (Fig. 6A). This was plausible because these peripheral DEGs were mainly expressed in non-neuronal cells, mainly in brain resident T cells, microglia, and vascular cells, according to published scRNA-seq data from human caudate nuclei^11^ (Fig. 6B).

Using available striatal samples from the same R6/1 mice^25^ in which blood was examined (Fig. 5), we confirmed that the upregulation of *Dse*, *Ifitm1* and *Irf7* in the striatum was much more consistent compared to blood in the mouse model, with *Irf7* being the most changing gene (Fig. 6C). Then, we evaluated whether the striatal expression of these genes was correlated with both the peripheral levels and the phenotype. However, we did not observe any correlation with motor impairment, including *Irf7* (Fig. 6D, unadjusted *p*-value > 0.05). Notably, the expression of *Irf7* and *Rsad2*, two genes also expressed in the human and mouse brains, was mutually correlated within each compartment (either in striatum or blood) (Fig. 6E). In contrast, the expression of each gene was not correlated across compartments (Fig. 6E), strengthening the view that brain and peripheral alterations of the same genes were largely dissociated. A possible reason is that these genes are likely to be expressed under distinct regulatory mechanisms, influenced by tissue context. Taking into account that the transgenic R6/1 strain is an aggressive model which use could be an important limitation of our study, slow progressive knock-in-based strains that reproduce both the mHTT production and the loss of a functional HTT allele can improve brain and peripheral correlations. Also, they can facilitate comparisons between patients and mouse models, as the manifested phenotype is more similar to the human pathology. However, in contrast to patients^18,52,53^, neither the transgenic nor knock-in HD mouse models develop a consistent neuroinflammatory response in the striatum (e.g., gliosis and microglia activation)^11,25,26^, which may limit the translation of our results from mice to humans. Nonetheless, the mild but significant upregulation of at least three genes involved in immune response in the striatum of a HD mouse model is of interest, which is in agreement with the documented increase of interferon-responsive genes in the striatum of R6/2 and zQ175DN mouse models^11^. These observations may be explained by still undisclosed roles for these genes in the dysfunction of the basal ganglia in these animals, which may have a more general impact on the correct functioning of the corticostriatal circuitry. For example, complement proteins secreted from locally activated microglia can provoke synaptic loss in presymptomatic stages that can lead to cognitive impairment^54^.

## Conclusions

Our results from own single-cell transcriptomics indicated that no clear / substantial transcriptional alterations were accumulated in cellular subpopulations to justify cell sorting procedures for prognostic procedures. This result was supported by comparisons with previous bulk transcriptomics reports aimed at obtaining transcriptional-based biomarkers from peripheral blood of HD patients. As our pools consisted of samples from few donors, we attempted to compensate the lack of correlative evidence based on clinical data by using our previously published phenotypical data from mouse models inbred under controlled genetic and environmental conditions, thus minimizing confounding factors. Despite species-specific divergences that can be an important limitation of this approach, we were able to find moderate correlations with the rotarod performance and the lymphocytic expression of *Irf7* gene, which striatal counterpart was prominently upregulated in the mutant mice. However, we found that *Irf7* expression was dissociated between striatum and blood of the same animals, undermining the use of peripheral markers to effectively infer the disease stage in the brain. More exhaustive screens in phenotypically characterized mice may reveal more and better correlated genes, which would serve as potential candidates for their subsequent assessment in humans.

## Supplementary Information

Below is the link to the electronic supplementary material.

Supplementary Material 1

## Data Availability

The scRNA-seq data can be downloaded from ArrayExpress using the accession number E-MTAB-15129.
