# Supplementary material for: Single-cell transcriptomics and mouse model phenotyping for biomarker screen of peripheral blood in Huntington’s disease
Source: Sci Rep. 2026 May 26;16:23980. doi: 10.1038/s41598-026-54412-4 (PMC13434588; doi:10.1038/s41598-026-54412-4)
Supplement: Supplementary file 1 [file 41598_2026_54412_MOESM1_ESM.pdf]

Supplementary Figure 1. Cell filtering in the scRNA-seq assays. *A*, The selected window for cell sorting was the same for all samples. *B*, Violin plots showing the parameters of nFeature_RNA (number of unique UMIs / cell), nCount_RNA (total number of reads / cell) and percent.mt (% of mitochondrial reads) for each pool of samples after filtering (see Materials and Methods).

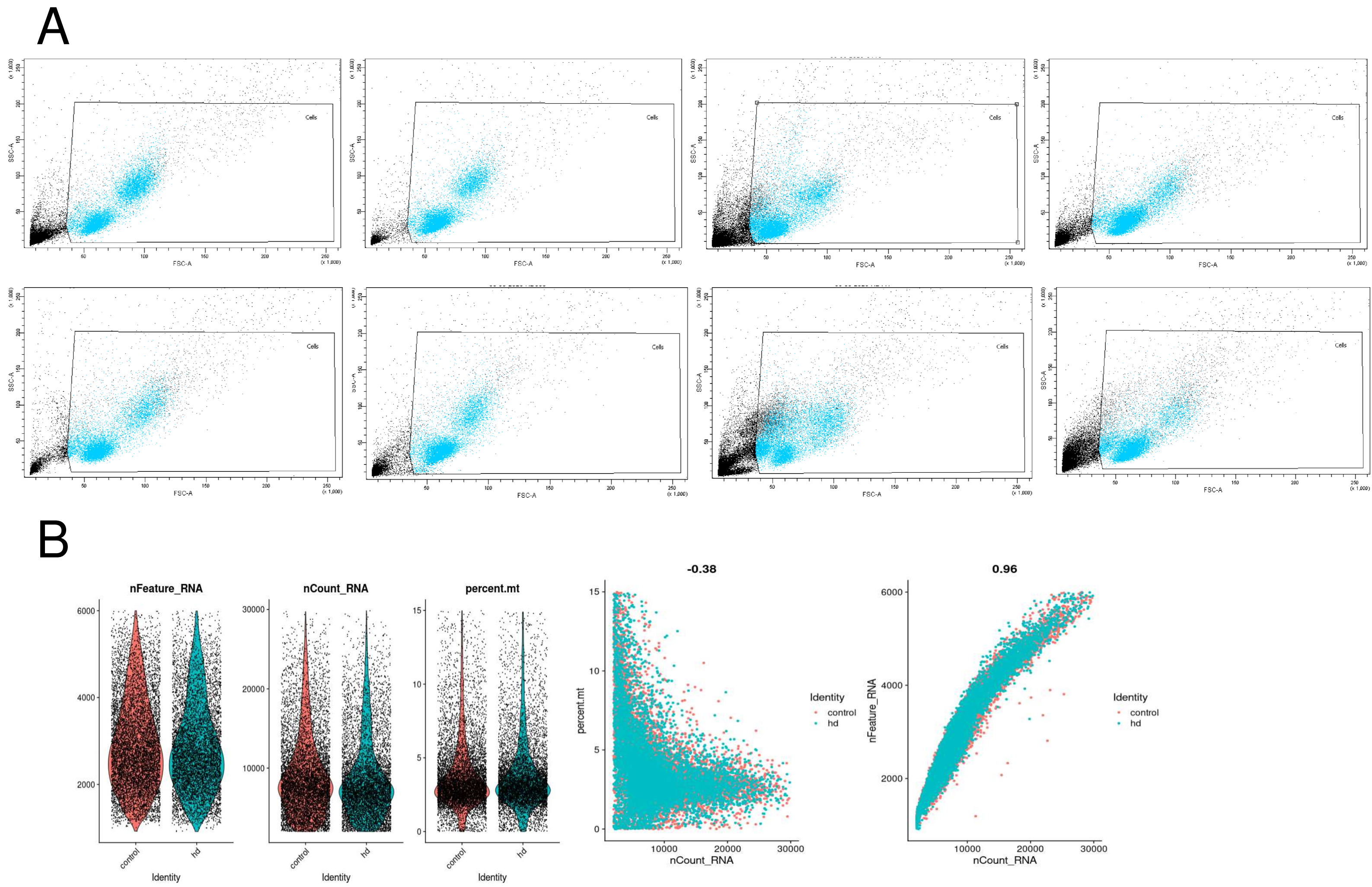

Supplementary Figure 2. Different contribution of donors to cellular clusters in SC2. Left, UMAPs showing the PBMC subpopulations. Right, UMAP with the distribution of *XIST* expression.

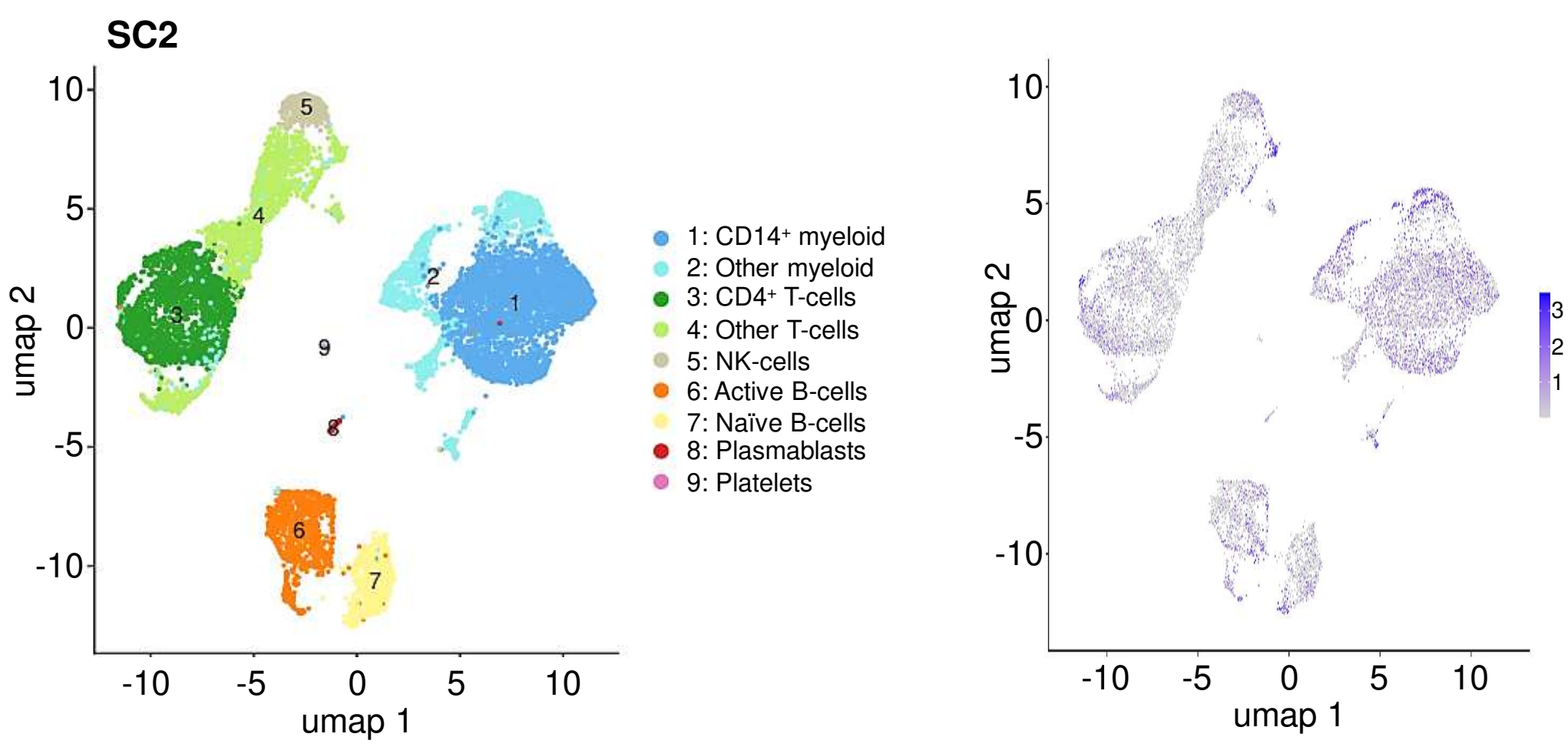

Supplementary Figure 3. Correlation between gene expression and motor performance in the R6/1 mouse model. *A-B*, Heatmap of Spearman correlation coefficients (ρ) between the examined transcripts in blood (*A*) and striatum (*B*) from R6/1 mice and rotarod performance at 11 (11w) and 13 (13w) weeks.

A

	11w	13w
Dse	0.17	0.05
Ifit2	0.06	0.03
Ifitm1	0.20	0.08
Irf7	-0.11	-0.01
Mx1	0.27	0.30
Rsad2	0.10	0.14
Samsn1	0.24	0.13

-1 ρ 1

B

	11w	13w
Dse	0.19	0.18
Ifitm1	-0.16	-0.18
Irf7	0.04	-0.03

-1 ρ 1
